# Exploring Antifouling Activity of Biosurfactants Producing Marine Bacteria Isolated from Gulf of California

**DOI:** 10.3390/ijms21176068

**Published:** 2020-08-23

**Authors:** Monserrat Alemán-Vega, Ilse Sánchez-Lozano, Claudia J. Hernández-Guerrero, Claire Hellio, Erika T. Quintana

**Affiliations:** 1 Instituto Politécnico Nacional, Centro Interdisciplinario de Ciencias Marinas, Av. Instituto Politécnico Nacional S/N. Col. Playa Palo de Santa Rita, 23096 La Paz, Baja California Sur, Mexico; monsealeman93@gmail.com (M.A.-V.); isanchezl1400@alumno.ipn.mx (I.S.-L.); 2 Univ Brest, CNRS, IRD, Ifremer, LEMAR, Institut Universitaire Européen de la Mer, F-29280 Plouzané, France; 3 Instituto Politécnico Nacional, Escuela Nacional de Ciencias Biológicas, Prolongación de Carpio y Plan de Ayala s/n, Col. Santo Tomás, Alcaldía Miguel Hidalgo, C.P. 11340 Ciudad de Mexico, Mexico; equintanac@ipn.mx

**Keywords:** adhesion, biofilm, biosurfactants, environmentally friendly antifouling, field assays, *Bacillus niabensis*, *Ralstonia* sp.

## Abstract

Biofouling causes major problems and economic losses to marine and shipping industries. In the search for new antifouling agents, marine bacteria with biosurfactants production capability can be an excellent option, due to the amphipathic surface-active characteristic that confers antimicrobial and antibiofilm activities. The aim of this study was to evaluate the antifouling activity of biosurfactants producing marine bacteria from the Gulf of California. The cell free culture supernatant (CFCS) of *Bacillus niabensis* (S-69), *Ralstonia* sp. (S-74) (isolated from marine sediment) and of *B. niabensis* (My-30) (bacteria associated to the sponge *Mycale ramulosa*) were screened for production of biosurfactants (using hemolysis and drop collapse test, oil displacement and emulsifying activity). The toxicity and antifouling activity were evaluated against biofoulers (bacteria forming biofilm and macrofoulers) both in laboratory and field assays. The results indicate that all bacteria were biosurfactant producers, but the higher capability was shown by *B. niabensis* (My-30) with high emulsifying properties (E24) of 71%. The CFCS showed moderate toxicity but were considered non-toxic against *Artemia franciscana* at low concentrations. In the antifouling assay, the CFCS of both strains of *B. niabensis* showed the best results for the reduction of the biofilm formation (up 50%) against all Gram-positive bacteria and most Gram-negative bacteria with low concentrations. In the field assay, the CFCS of *B. niabensis* (My-30) led to the reduction of 30% of biofouling compared to the control. The results indicate that the biosurfactant produced by *B. niabensis* (My-30) has promising antifouling activity.

## 1. Introduction

The Gulf of California is an ecosystem of rich biodiversity with abundant biological resources [1]. From this region, some bacterial strains with interesting biological properties have been successfully isolated [2]. Within these activities, antibiofilm and antifouling potency can be mentioned [3,4].

In marine environments, epibiosis or biofouling processes start with biofilm formation, and then the settlement of other sessile organisms such as macroalgae and/or invertebrates [5,6]. Biofouling on immersed man-made structures leads to operational difficulties in numerous industrial sectors such as shipping, energy production, fishing or aquaculture, ranging from corrosion to biodeterioration. Moreover, it alters the electrical conductivity of materials, increases the weight or volume of man-made immersed structures and causes economic losses [7,8]. In order to delay or inhibit biofilm formation and settlement of organisms, usually surfaces are treated with paint formulation containing toxic substances such as copper, zinc, lead or other biocides. These compounds are very effective in the control of biofouling, but their mode of action is based on toxicity [9,10,11]. For this reason, the development of environmentally-friendly solutions for the control of biofouling is important. Natural antifouling (AF) agents from marine bacteria have been proposed as one of the best possible alternative approaches to replace current biocides in paints [12,13]. 

In recent years, it has been demonstrated that bacterial biosurfactants showed interesting antibacterial and antibiofilm activities against several pathogens [14,15,16,17]. Biosurfactants are amphipathic surface-active compounds that are synthesized by numerous microorganisms. The interest of these compounds is due to their low toxicity, their biodegradability and their environmental compatibility that allow wide biotechnological applications in different areas such as the bioremediation, food, cosmetic, agronomic, pharmaceutical and antifouling industries [18,19,20]. Biosurfactants significantly reduced the surface tension property and thus confers activity as anti-adhesives and antimicrobial/antibiofilm agents [21]. Studies have highlighted that marine microorganisms are capable of producing novel biosurfactants with excellent bioactivity [21,22,23,24,25]. Moreover, these novel biosurfactants isolated from bacteria often prove especially effective in the disruption of biofilm [22,23,26]. Bacteria associated with macro-organisms can produce bioactive biosurfactants, in some cases with maximum stability and exceptional emulsifying capability [27], and can be considered as a promising source for novel AF agents. For example, lipopeptides and glycolipid biosurfactants produced by marine actinobacteria sponge-associated or by coral associated bacteria showed significant antibiofilm activity [28,29]. 

The aim of this study was to evaluate the antifouling activity of biosurfactants produced by marine bacteria isolated from the Gulf of California. The antifouling activity of the crude biosurfactants was evaluated both in the laboratory and field assays with the goal to search for a nontoxic alternative to reduce biofouling on marine structures. 

## 2. Results

### 2.1. Isolation and Identification of Marine Bacteria

The isolates from marine sediments and sponge-associated bacteria evaluated in the present study were identified to genus and species levels by 16S ribosomal sequencing and phylogenetic analysis “barcoding”. Both the associated sponge bacteria My-30 and the strain isolated from sediment S-69 were identified as *Bacillus niabensis*, but the morphology characteristics were different between strains. The third strain isolated from marine sediment (S-74) was identified as *Ralstonia* sp. (Table 1). The phylogenetic trees of each strain are shown in the supplementary information (Figures S1–S3).

### 2.2. Screening for Production of Biosurfactants

**Figure 1:**
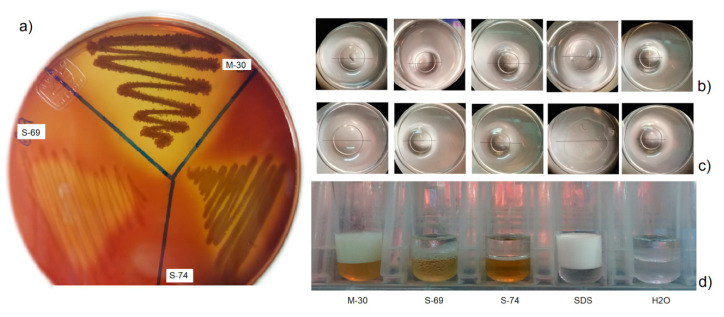
Evaluation of the effect of biosurfactants of marine bacteria isolated from the Gulf of California. Supernatants of *Bacillus niabensis* (My-30), *Bacillus niabensis* (S-69), *Ralstonia* sp. (S-74), (controls: SDS 10% and H_2_O). (**a**) Hemolytic activity, (**b**) drop collapsing in olive oil, (**c**) drop collapsing in mineral oil and (**d**) emulsification properties at 24 h.

The cell-free culture supernatant (CFCS) from each strain was screened for the production of biosurfactants following 4 classical methods: hemolysis activity, drop collapsing, oil displacement and emulsification properties. The three bacteria showed different grades of positive results (Table 2, Figure 1). The sponge-associated bacteria *Bacillus niabensis* (My-30) showed the best activity with similar results to the positive control (SDS 10%) in the collapsing drop test and emulsification properties with high stability for 24 h even greater than the control SDS (10%) (Table 2, Figure 1).

### 2.3. Toxicity of the Cell Free Culture Supernatants

The toxicity evaluation of the cell-free supernatants after 24 h incubation is summarized in Table 3. At low concentrations, the supernatants were non-toxic and did not affect the swimming behavior nor the survival rate of *Artemia franciscana*. At high concentrations, the survival rate was reduced by 50% (LC_50_ ˃ 250 µL mL^−1^). The toxic compounds TBTO and CuSO_4_ showed LC_50_ values of 8.5 and 6.2 µg mL^−1^, respectively.

### 2.4. Evaluation of the Antifouling Activity 

#### 2.4.1. Antibacterial Bioassay

The activity of cell free culture supernatants (CFCS) for the growth inhibition of marine bacteria involved in biofilm formation (expressed as Minimum Inhibitory Concentration, MIC) is summarized in Table 4. Supernatants of *Bacillus niabensis* (both strains My-30 and S-69) were the most active with MIC values between 1% and 2% *v*/*v*. The most susceptible bacteria towards the bacterial supernatants were *Bacillus subtilis* (Bs), *Micrococcus* sp. (Msp1) and *Sagittula stellata* (Ss). However, CFCS at low concentrations did not inhibit the growth of *Pseudoalteromonas* Sp.1, Sp.2 or *Vibrio* sp., and thus, it is necessary to use concentrations up to 10% *v*/*v* to achieve inhibition.

#### 2.4.2. Inhibition Bacterial Adhesion Bioassay

**Figure 2:**
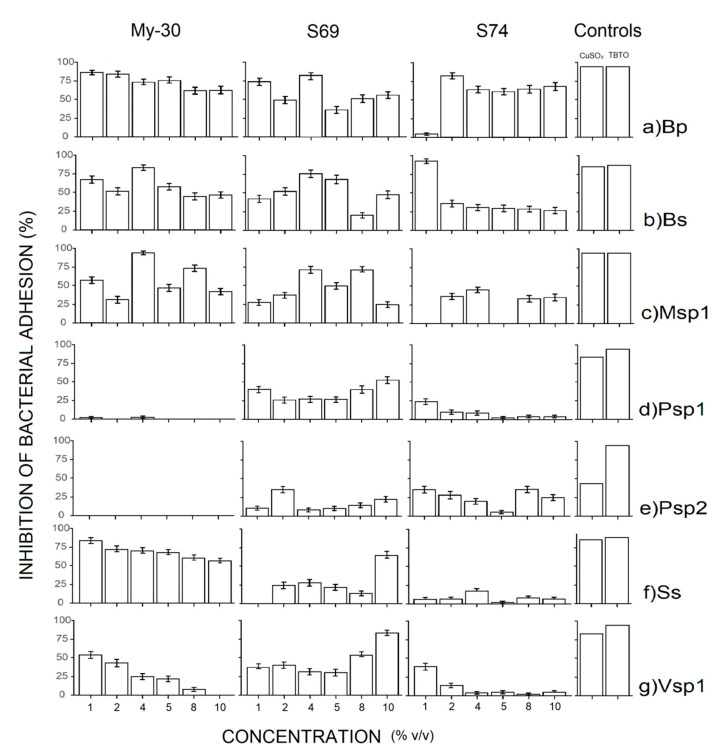
Assessment of the efficiency of bacterial supernatants of *Bacillus niabensis* (My-30 and S-69) and *Ralstonia* sp. (S-74) for the inhibition of bacterial adhesion, (**a**) Bp (*Bacillus pumilus*), (**b**) Bs (*Bacillus subtilis*), (**c**) Msp1 (*Micrococcus* sp. 1), (**d**) Psp1 (*Pseudoalteromonas* sp.1), (**e**) Psp2 (*Pseudoalteromonas* sp.2), (**f**) Ss (*Sagittula stellata)* and (**g**) Vsp1 (*Vibrio* sp.1). Controls CuSO_4_ (Cu) and TBTO were assayed at 10 µg mL^−1^.

The results of the inhibition of bacterial adhesion showed that all free-cell supernatants tested inhibited the adhesion of at least 4 biofilm forming bacteria strains (Figure 2). The strains most susceptible to the supernatants were the Gram-positive *Bacillus pumilus* (Bp), *B. subtilis* (Bs) and *Micrococcus* sp.1 (Msp1). While the Gram-negative bacteria *Pseudoalteromonas* sp.1 and *Pseudoalteromonas* sp. 2. were more resistant and the supernatants S-69 and S-74 only can inhibit the bacterial adhesion below 50% (Figure 2).

The sponge associated *Bacillus niabensis* (My-30) displayed very potent activity against *Bacillus pumilus* (Bp), *B. subtilis* (Bs), *Micrococcus* sp. 1, *Sagittula stellata* (Ss) and *Vibrio* sp.1 (Vsp1), and inhibited the adhesion between 50% and 80% when used at low concentrations (1% to 2% *v*/*v*), but was not active against *Pseudoalteromonas* sp.1 and sp. 2. The supernatants of *B. niabensis* isolated from sediment (S-69) and *Ralstonia* sp. (S-74) were able to inhibit the adhesion of all bacteria, but it is necessary to use a higher concentration to inhibit all strains (2% to 10% *v*/*v*). The toxic compounds TBTO and CuSO_4_ inhibited the bacterial adhesion up to 80% in most cases.

### 2.5. Antifouling Assay in Natural Conditions

**Figure 3:**
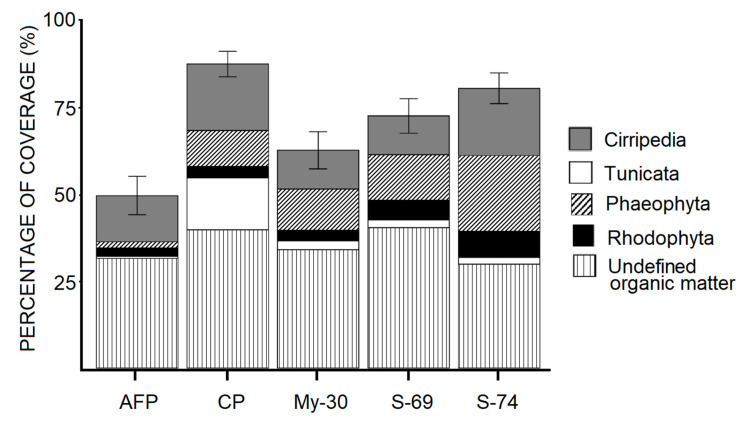
Percentage of epibiont coverage in different treatment plates after 130 days of exposure in the field (Marina La Paz). Antifouling Paint (AFP), control plate with formulated paint (CP), plates with *Bacillus niabensis* cell free culture supernatant (CFCS) (My-30, S-69) and plate with *Ralstonia* sp. CFCS (S-74).

The results of the coverage percentages for each group of organisms settled in the plates after 130 days of exposure in La Paz Marina are presented in Figure 3. The control plate ((CP) with formulated paint) showed 87% coverage (37% undefined organic matter and 50% organisms), and the organisms that were settled were identified as barnacles (Cirripedia), macroalgae (Phaeophyceae and Rhodophyceae) and ascidians (Tunicates). The plate with commercial AF paint showed 49% coverage, but only 19% of which was covered by settled macroorganisms. The plates painted with the supernatants showed a reduction in the biofouling coverage. The supernatant of *B. niabenins* (My-30) was the more active and led to a significant reduction in the coverage of 30% compared to control (CP).

**Figure 4:**
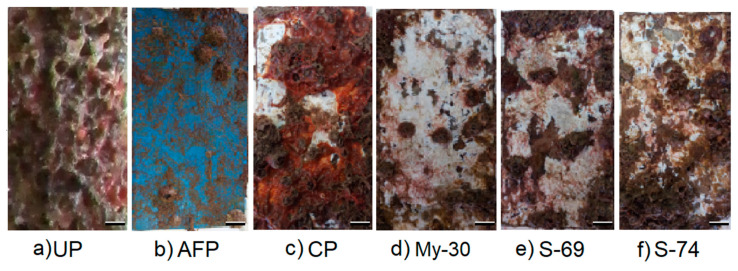
Settlement of organisms in the different treatment after 130 days of immersion in field conditions (Marina of La Paz). (**a**) Structure PVC unpainted (UP), (**b**) Antifouling Paint (AFP), (**c**) control plate with formulated paint (CP), (**d**) supernatant of (My-30), (**e**) supernatant of (S-69) and (**f**) supernatant of (S-74). (Scale bar: 1 cm).

After 130 days of immersion of the plates in the marina of La Paz, the unpainted PVC structure (UP) that supported the plates were fully covered by epibionts (Figure 4a), while the commercial antifouling paint (AFP) inhibited the settlement of tunicata and macroalgae (Figure 4b). Similar to AFP, the three CFCSs (My-30, S-69 and S-74) (Figure 4d–f) inhibited the settlement of ascidia *Distaplia stylifera* (Tunicata) in comparison to the control (CP) (Figure 4c), but only My-30 and S-69 inhibited the barnacle settlement, demonstrating the effectiveness of these supernatants as antifoulants.

## 3. Discussion

The use of toxic agents for the formulation of antifouling paints (such as TBTO (now banned) or heavy metals such as copper or zinc) to avoid the biofouling on immersed man-made structures in the sea has detrimental environmental impacts. It is thus urgent to develop innovative alternatives with low impact on the environment [30]. In the biofouling process, one of the first steps consists of the bacterial adhesion, which involves hydrophobic interactions and hydrophobic adhesins [31]. For several years, it has been known that biosurfactants are disruptors of hydrophobic interactions on the attachment of a series of marine and estuarine isolates [32]. In recent years, the biosurfactants study has increased and their potential as an effective alternative in the control of biofilms was highlighted [14,22,33]. Among the advantages compared to other chemical or antibacterial agents, we can list low toxicity, biodegradability, biocompatibility and effectiveness under various environmental conditions [23,34,35]. The Gulf of California is a source of diverse marine bacteria taxa [36], and it has been demonstrated that some bacteria have antifouling activity [3,4]. With this view, the objective of this work is to evaluate the production of free cell supernatant biosurfactant from three bacteria isolated in different environmental localities (marine sediment and sponge-associated) and their antifouling activity both in laboratory (by evaluating inhibition of growth and adhesion of bacteria involved in biofouling process) and in field (activity in a paint matrix).

### 3.1. Isolation and Identification of Marine Bacteria with Antifouling Activity

The marine bacteria utilized in this work were identified by phylogenetic analysis of 16S rDNA sequence. The strains My-30 and S-69 corresponding to *Bacillus niabensis*, Gram-positive bacteria described for the first time in 2007, the isolates from cotton-waste composts for mushroom cultivation were named from the acronym NIAB of the National Institute of Agricultural Biotechnology in Korea [37]. In the marine environment, *B. niabensis* has been isolated, associated with the soft coral *Alcyonium digitatum* from the Baltic Sea [38]. 

It not surprising that isolates My-30 and S-69 from different environments were finally the same species: My-30 was associated with sponge *Mycale ramulosa* isolated in Pichilingue locality, while S-69 was isolated in marine sediment in Punta Arena de la Ventana locality. Members of the genus *Bacillus* are ubiquitous in nature and are abundant representatives of both marine sediments [39] and associated with marine organisms (algae, ascidia, sponge) [3,40,41,42]. Nevertheless, the morphological and bioactive characteristics are different between strains. 

The strain S-74 was identified as Gram-negative bacteria *Ralstonia* sp. Strains of this genus have been isolated from plants [43], marine sediment [44] and estuary ecosystems [45]. 

### 3.2. Screening for Production of Biosurfactants

The biosurfactants are produced in nature as a diverse group of secondary metabolites comprising glycolipids, lipopeptides, lipoproteins, fatty acids, neutral lipids and polymeric phospholipids [46]. Isolated marine Bacillus produced structurally diverse classes of secondary metabolites including biosurfactants that exhibit potent surface and emulsifying properties [47] such as polypeptides and cyclic lipopeptides surfactins, iturins and fengycins [47,48].

Within the methodologies used to detect the production capacity of biosurfactants in the bacterial supernatant, the more sensitive described in the literature were the drop collapsing and oil displacement. In the case of hemolytic activity, not all biosurfactants have this activity, for this reason, we used it as a complementary test [49,50]. In our study, supernatants of My-30, S-69 and S-74 showed, indeed, a different degree of ability to produce biosurfactants.

Of the three strains, My-30 *B. niabensis* (sponge-associated) had the higher capability to produce biosurfactants with emulsifying properties, with the largest zone of hemolysis and emulsification efficient after 24 h (E24) of 71%. This result can be considered promising if compared with the values obtained from *Bacillus subtilis* N10 (E24 = 70%) [51], and the compound Surfactin (the most powerful lipopeptide surfactants) with E24 = 67.6% [52]. Three Surfactin homologous were purified from supernatant cultures of *B. niabensis* CWBI-B11569 3 [53].

In the case of *Ralstonia* sp. (S-74), good results for the drop collapsing test (5.2 mm) were obtained as they were similar to the control SDS (10%). Studies with *Ralstonia picketti* demonstrated its capability for crude oil degradation over 80% in 20 days of incubation [54]. This genus produced rhamnolipids that are biosurfactants belonging to glycolipids [55]. Some strains of *Ralstonia* are able to degrade aromatic pollutants [56,57], and other compounds such as trichloroethene, phenol, polychlorinated biphenyl (PCB), biphenyl, benzene and, more recently, a strain that can degrade 3,5,6-trichloro-2-pyridinol (TCP) [58,59,60,61,62,63].

### 3.3. Toxicity Assay

In order to protect the marine environment, it was necessary to ensure that the supernatants that would be evaluated in the different bioassays and fields were not toxic. The supernatants had LC_50_ values ranging from 260 to 330 µL mL^−1^. These values can be considered moderately toxic in accordance to the classification of Fernández-Calienes et al. [64]. However, it is important to mention that the concentrations required for the inhibition of organisms involved in biofouling were below 100 µL mL^−1^ and that these concentrations were non-toxic to *Artemia franciscana.* In the case of the biocides, TBTO and CuSO_4,_ showed high toxicity with LC_50_ of 8.7 and 6.2 µg mL^−1^, respectively; these values are considered extremely toxic [64]. The ecological effect of bioaccumulation of TBT in marine species and possible human health risk caused its ban. The cooper is still used, but its toxic action led to the development of other compounds with AF activity which were nontoxic to the marine environment [65]. The toxicity is an important factor when proposing candidates for AF agents, and this can be from low to nontoxic [66].

### 3.4. Evaluation of the Antifouling Activity

#### 3.4.1. Antibacterial Bioassay

The supernatants (CFCS) of *B. niabensis* My-30 and S-69 showed potent antibacterial activity against Gram-positive bacteria *Bacillus pumilus*, *B. subtilis* and *Micrococcus* sp., but showed less activity against Gram-negative *Pseudoalteromonas* sp1, sp2 and *Vibrio* sp. These results are similar to the ones found by Fernandes et al. [67] where surfactants produced by *Bacillus* were more active against Gram-positive bacteria than Gram-negative. The genus *Bacillus* has the capability to produce lipopeptide types of biosurfactants with potent antimicrobial activity [68,69]. Lipopeptides with strong antibacterial activity have the ability to destroy the bacterial cell membranes by detergent-like action on cell membranes. This considerably reduces the development of bacterial resistance, and thus provides an excellent alternative method for the reduction in bacteria [69]. 

Lipopeptides produced by *B. amyloliquefaciens* M1 had a broad spectrum of action, including antibacterial activity against the Gram-negative pathogenic *Vibrio* spp. with multidrug-resistant profiles [69]. In the present work, the CFCS of the 2 strains of *B. niabensis* were able to inhibit the Gram-negative bacteria *Sagittula stellata* at low concentrations.

The CFCS of *Ralstonia* sp S-74 showed good activity against Gram-positive bacteria *Bacillus* sp. and *Micrococcus* sp. *Ralstonia* genus, which had the capability to produce rhamnolipids [55], whose are considered successfully as antimicrobial agents with antibacterial activity against Gram-positive and negative bacteria [70].

#### 3.4.2. Inhibition Bacterial Adhesion Bioassay

The bacterial biofilm development is a significant problem in different environments (medical, food, fouling) and is difficult to treat due to their resistance to antimicrobial treatment [22,71]. In the present study, the promising results in the inhibition of marine bacterial adhesion are interesting, because in accordance with the International Antimicrobial Council (2015) [72], the demand for antimicrobial coatings is growing, with a market volume estimated to 589.8 kilo tons by 2020. For this reason, it is a real challenge for the industry. The production of biosurfactants with the potential to inhibit the bacterial adhesion could have high costs in production and purification [73]. Nevertheless, it has been suggested that for environmental purposes it is possible to use crude biosurfactants [74]; thus, this would significantly reduce the cost of production and would be a great advantage for economic viability. The advantage of growing bacteria to produce these natural compounds is that controlled cultivation can increase the yields of biosurfactant production. However, studies are needed to optimize production and to find new culture media (such as raw materials) in order to reduce costs and implement relatively simple processes [75].

*Bacillus niabensis* (My-30) isolated from marine sponge, when used at low concentrations, demonstrated high activity in the inhibition of bacterial adhesion against Gram-positive and Gram-negative bacteria (with the exception of *Pseudoalteromonas* sp.). Bacteria of the *Bacillus* class have demonstrated inhibitory activity in high ranges and some inhibiting most biofilm forming strains [3,42,76,77,78]. It is of great interest that My-30 showed good inhibition of bacterial adhesion as well as a very high capacity to produce biosurfactants. *Bacillus* genus is known for the production of different types of biosurfactants, and they are excellent inhibitors of microbial adhesion thanks to a mode of action based on the disruption of the preformed bacterial biofilm [79].

### 3.5. Antifouling Assay in Natural Conditions

The addition of CFCS of *Bacillus niabensis* (My-30) improves the efficiency of the formulated paint by 30% (inhibition of attachment). Although there are several studies that add natural products [80] or extracts from marine organisms to paint formulation [81,82], microbial extracts are rarely used [83,84]. Previously, supernatants of marine bacteria isolated from algae and nudibranchs have been tested; only the latter showed significantly higher activity than the controls [85]. The incorporation of *Pseudomonas aeruginosa* (bacteria) in epoxy paints inhibited the macrofouling even up to 60 days immersed in natural seawater; the compound responsible for the antifouling activity was a bacteriocin isolated from the bacteria [84]. For our study, the only CFCS that led to a significant difference to the control (CP) and commercial antifouling paint (AFP) was sponge-associated *Bacillus niabensis* (My-30), and the activity was conserved during 130 days. The immersion time of the experimental plates ranged from 45 to over 185 days, and this time allowed the integrity of the paint and the strength of the extract or compound in the paint to be studied [81,82]. However, after 185 days of immersion, many species die due to the competition for food and it is possible that extracts or compounds tested are completely leached out [82]. In our case, the experiment lasted 130 days because complete coverage of the unpainted structure (UP) was observed and the experimental paint began to leach.

The color of the substratum is a factor that can affect the formation and settlement of biofouling communities; for that reason, in ecological experiments, it is important that the experimental and control plates have a similar color [86]. In our study, although the AF commercial paint was blue, the control (CP) and experimental plates were painted in the same color (white), and the results are comparative. The color on the surface (especially black and red) can affect the quantities of radiant energy absorbed or reflected, and this has an effect on the diversity and composition in the biofilm community and the subsequent settlement of marine invertebrates [87]. The surface roughness is another factor to consider, because it can reduce the bond strength of biofilms and reduce the settlement of algae spores [88,89]. In this study, it was not possible to determine the roughness of the plates after painting; further research is needed if we want to evaluate this. 

The results suggested that the crude supernatant of My-30 was capable of controlling the initial sedimentation of microbes and avoiding the settlement of barnacles (Cirripedia) and the ascidia *Distaplia stylifera* (tunicate). It is important to point out that these results are very promising because the colonization of these organisms poses significant problems for bivalve culture and the maritime industry in the Bay of La Paz [90]. However, in order for the crude supernatant of My-30 to be used commercially, several strategies must be implemented, such as controlling the leaching rate of the compound from the paint and developing a coating that releases the compound as slowly as possible to the surface [8]. In addition to identification, it will be necessary to work on the optimization and large-scale production of the biosurfactant or explore the possibility of using raw biosurfactants to avoid the cost of separating the compounds [73,74].

## 4. Materials and Methods

### 4.1. Sample Collection and Isolation of Marine Bacteria

#### 4.1.1. Collection of Native Marine Bacteria Involved in Biofilm Process

The 7 native marine bacteria used in this research were isolated previously from La Paz Bay, B.C.S., Mexico [91] and belong to the collection of Laboratory of Microbiology and Molecular Biology of IPN-CICIMAR. The strains used for this work were 3 Gram-positive bacteria: *Bacillus pumilus* (Bp), *B. subtilis* (Bs), *Micrococcus* sp.1, (Msp) and 4 Gram-negative: *Pseudoalteromonas* sp.1 (Psp1)*, Pseudoalteromonas* sp.2 (Psp2), *Sagittula stellata* (Ss) and *Vibrio* sp.1 (Vsp1). All these strains are involved in the biofilm process [91].

#### 4.1.2. Chemicals

The chemical used for the identification of marine bacteria were molecular biology grade. All other chemicals used within this research project were analytical grade, obtained commercially.

#### 4.1.3. Isolation and Identification of Marine Bacteria

Strain My-30 was previously isolated from the sponge *Mycale ramulosa* Carballo & Cruz-Barrazo (2010). Specimens were collected by snorkeling in Pichilingue locality (24°17′ N–110° 20′ W) at a depth of 3 m, placed into sterile plastic bags, cooled on ice and transported immediately to the laboratory. In sterile conditions, 5 g sponge tissue was washed with 70% ethanol and subsequently with filtered sterile sea water, and ground in a mortar. Serial dilutions (10^−1^ to 10^−5^) were prepared, and 100 µL aliquots were spread onto plates of Difco Marine Agar (in triplicate) and incubated at 35 °C for 24 h.

Strains S-69 and S-74 were isolated from the marine sediment of Punta Arena de La Ventana locality (24°03′ N–109° 49′ W). Sediment samples were collected by scuba diving at a depth of 5 m using a sterile plastic tube modified as a sediment sampler. The isolation was realized with the dry/stamp (DS) method [92]. The sediment was dried overnight in a laminar flow hood. Posteriorly, the sediment was taken with sterile foam (2 cm diameter) as a seal and stamped in the culture medium plate eight times, like a serial dilution effect. The plates were incubated at 35 °C for 24 h.

The bacteria colonies were isolated and characterized based on morphology and Gram staining. Bacteria were identified by partial sequencing of 16S rRNA. From the culture, genomic DNA was extracted and then purified. PCR was performed using specific oligonucleotides for the 16s rDNA gene [93]. Each PCR reaction was run together with positive controls (genomic DNA mixture bacterial) and negative controls (oligonucleotides + sterile water without DNA, sterile water + DNA without oligonucleotides). The amplification products were separated by 2% agarose gel electrophoresis and visualized on a UV light transilluminator. Bands were cut into samples that had specific amplification, and the DNA of each band was purified using the Zymo Clean Gel recovery kit. The purified DNA from each band was sent to the IBT Sanger sequencing service (UNAM). The sequences were analyzed in BLASTN and the phylogenetic analysis or “Barcoding” (alignment of the sequences obtained with the reference sequences corresponding to the bacterial genus determined by the analysis in BLASTN with MUSCLE) was performed. The resulting alignments were analyzed with the Neighbor-Joining (NJ) method and the model of Tamura-Nei parameters, with the statistical support of 1000 replicas of “Bootstrap”.

### 4.2. Isolation of Bacterial Supernatant

The cell free culture supernatants (CFCS) were obtained according to the methodology described by Sayem et al. [42] with some modifications. The isolated bacteria were cultivated on marine agar in petri dishes (1.8% agar) and incubated for 48 h at 35 °C. Then, the culture was standardized to a turbidity of 1 (OD, 600 nm) in saline solution (2%), subsequently inoculated in marine broth at 10% (in triplicate) and incubated at 35 °C for 5 days with agitation of 150 rpm. The culture was centrifuged at 6000 rpm for 20 min in order to separate the cell pellets from the medium. Supernatants were then filtered (Minisart filter with a pore size of 0.2 µm). In order to confirm the absence of cells in the filtrates, 100 µL was deposited on agar plates (in triplicate) and cultured for 24 h at 35 °C. The cell free culture supernatants (CFCS) were further used for drop collapse, oil spreading, emulsification assay, toxicity assay and antifouling assay.

### 4.3. Primary Screening of Strains for Biosurfactant Production

#### 4.3.1. Hemolytic Activity

For primary screening of surfactant-producing strains, clearance in blood agar plates was used as an indicator. The hemolytic activity was evaluated using a modified version of protocol Mulligan et al. [94]. The experiment was run in triplicate. The strains were inoculated in blood agar containing 5% (*w*/*v*) peptone, 3% (*w*/*v*) yeast extract, 5% (*w*/*v*) NaCl and 5% (*v*/*v*) sheep blood. After 48 h at 35° C incubation, the plates were examined for colony growth with a zone of clearance around the colony. The biosurfactants had the ability to induce lysis of the blood erythrocytes [27,50].

#### 4.3.2. Drop Collapsing Test

The assay was carried out as described by Youseef et al. [49]. An amount of 2 µL mineral oil was applied to the well regions delimited on the covers of 96-well microplate. After one hour, 5 µL of the supernatant was transferred to the oil coated regions, and the drop size was observed 1 min later. The experiment was run in triplicate. The result was positive when the drop diameter was at least 1 mm larger than that produced by distilled water (negative control). SDS (10%) was used as a positive control.

#### 4.3.3. Oil Displacement Test

The oil spreading assay was realized using the method described by Morikawa et al. [95]. Distilled water (20 mL) was added to a plastic cover Petri dish, followed by 20 µL oil (mineral and olive) to the surface of the water. An amount of 10 µL supernatant was added to the oil surface. The oil was displaced in the presence of the biosurfactant, showing an oil free clearance zone diameter. Distilled water was used as a negative control and SDS (10%) was used as a positive control. The experiment was run in triplicate.

#### 4.3.4. Emulsification Index

The supernatant emulsification activity was determined in accordance with Plaza et al. [55] method. In a tube, 2 mL supernatant and 2 mL toluene were added and mixed for 2 min, and then incubated for 24 h at room temperature in 3 replicates. The emulsification index (E.I.) was determined by the ratio of the height of the mix and the emulsificated layer, expressed in percentage. Distilled water and SDS at 10% were used, respectively, as a negative and positive control. Moreover, emulsion stability was determined by the volume of the emulsion layer at 0, 24 and 48 h [96].

### 4.4. Toxicity Assay

The toxicity of CFCS was evaluated using the brine shrimp lethality assay [97]. Experiments were run in 96-well flat bottom microplates (Costar 3596). An amount of 200 µL supernatant at different concentrations (1, 2, 4, 8, 16 and 32% *v*/*v*), diluted in sterile seawater (SSW), were added to the wells. Then 10 nauplii of brine shrimp (*Artemia franciscana* Kellogg 1906 Branchiopoda, Anostraca, Artemiidae) were added to each well and incubated at 30 °C for 24 h. Positive controls consisted of Tributyltin oxide (TBTO) solutions at 2.5, 4, 6, 8, 10 and 12 µg mL^−1^ and CuSO_4_ solutions at concentrations of 2, 4, 6, 8, 10 and 12 µg mL^−1^. As a negative control, wells with culture broth at the same concentrations of the supernatants were used. All experiments were performed in triplicate. The number of survivors in each well was counted under a stereoscopic microscope at 24 h, and the percentage of death was calculated. The lethal concentration at 50% (LC_50_) was calculated by a Probit Test in Stat Graphics program.

### 4.5. Evaluation of the Antifouling Activity in Laboratory

#### 4.5.1. Antibacterial Bioassay

Cell free culture supernatants (CFCS) of marine bacteria were used to evaluate the growth inhibition of bacteria involved in biofilm formation in accordance with the protocol of Sayem et al. [42] with slight modifications. The biofilm forming bacteria: *Bacillus pumilus* (Bp)*, B. subtilis* (Bs) *Micrococcus* sp.1 (Msp1), *Pseudoalteromonas* sp.1 (Psp1)*, Pseudoalteromonas* sp.2 (Psp2)*, Sagittula stellata* (Ss) and *Vibrio* sp.1 (Vsp1) were grown on marine agar at 35 °C for 24 h and further adjusted to a density of 1 × 10^8^ cells mL^−1^ in liquid media. The supernatants at concentrations of 1, 2, 4, 5, 8 and 10% *v*/*v* were added in 96-well flat bottom microplates (Costar 3596) and inoculated with the overnight culture bacteria (to complete 200 µL per well) (with 6 replicates each). The plates were incubated for 48 h at 35 °C. After recording the optical density at 620 nm in a microplate reader, the results were expressed as the minimum inhibitory concentration (MIC).

#### 4.5.2. Inhibition Bacterial Adhesion Bioassay

The bacterial adhesion assay was performed in accordance with Sayem et al. [42] and Hellio et al. [98]. The bacteria involved in biofouling were grown overnight on marine agar and adjusted to an optical density of 1.0 at 585 nm. Then, 200 μL of the bacterial suspension was inoculated in each well using a multichannel pipette. The covered plate was incubated for 48 h at 35 °C using horizontal shaking (150 rev min^−1^). The non-adhered bacteria were eliminated by 5 washings with NaCl solution (36 g L^−1^), and the adhered bacteria were stained with 200 μL crystal violet solution for 45 min. After staining, the plates were washed with sterile distilled water five times. The quantitative analysis of biofilm production was performed by adding 200 µL EtOH-Acetone solution (4:1). The OD level of crystal violet staining present in the solution was measured at 570 nm in a microplate reader (TECAN). TBTO and copper sulphate were utilized as controls. All assays were realized in 6 replicates. The results were expressed as the percentage of inhibition of bacterial adhesion.

### 4.6. Evaluation of the Antifouling Activity in Natural Conditions of Immersion

The antifouling paint matrix was made based on Acevedo et al. [81] methodology. Under laboratory conditions, colophony resin (27%) was dissolved in xylene (20%) and white spirit (20%); subsequently, oleic acid (6%), zinc oxide (16.2%) and calcium carbonate (10.8%) were added. The paint was filtered and separated, one part was used as a negative control (FP) and the other parts to include the 3 different supernatants (4% *v*/*v*).

Acrylic tiles (6 × 13 cm) were previously degreased and covered with 3 coats of paint (1.5 g per coat), with 24 h between each application. All the treatments were placed for triplicate testing in a PVC frame. Additionally, acrylic plates with formulated paint (CP), commercial antifouling paint (AFP) and part of the frame considered as the unpainted area (UP) in the same frame were used as controls. The frame was immersed in Marina La Paz inside La Paz Bay, Baja California Sur, México (24°09′18.6″ N–110°19′31.7″ W) at a 1.5 m depth for 130 days.

#### Coverage Percentage of Epibionts

A dot grid method (105 points were evaluated for each plate) [81] and the CPCe program were used to estimate the percentages of coverage of each group of organisms after 130 days of exposure in the Marina. The differences between each treatment were determined by a one-way analysis of variance test (ANOVA).

## 5. Conclusions

The associated *B. niabensis* sponge strain (My-30) produces biosurfactants with strong emulsifying properties. This crude biosurfactant, at a low concentration, led to a significant reduction in the formation of marine biofilm, with no recorded toxicity. In the evaluation under real immersion conditions, a 30% reduction in the attachment of macro-organisms was recorded. Thus, we can conclude that the biosurfactant producers *B. niabensis* (My-30) lead to promising antifouling activity. Further research into the purification, identification and large-scale, low-cost production of biosurfactants is needed to produce a paint available industry.

## Figures and Tables

**Table 1:** Marine bacteria isolated from sponge and sediment of coasts of Baja California Sur (with antifouling potential).

ID Strain	Species	Isolated Origin	Locality		GenBank Accession No.
My-30	*Bacillus niabensis*	Sponge *Mycale ramulosa*	Pichilingue	24°17′ N–110° 20′ W	MT887632
S-69	*Bacillus niabensis*	Marine sediment	Punta Arena	24°03′ N–109° 49′ W	MT887633
S-74	*Ralstonia* sp.	Marine sediment	Punta Arena	24°03′ N–109° 49′ W	MT887634

**Table 2:** Capability of marine bacteria isolated from the Gulf of California to produce biosurfactants with drop collapsing and emulsification properties.

	Biosurfactant Production Capability					Emulsification Properties (%)		
Bacteria	Hemolytic Activity	Drop Collapsing Test (mm)		Oil Displacement Test (Ø mm)		Emulsification Index (E.I.)	Emulsification Stability (E.S.)	
		Mineral Oil	Olive Oil	Mineral Oil	Olive Oil		24 h	48 h
My-30	+++	5.3	6.2	9	2	56	71	7.6
S-69	+	4.3	4.3	6	1.5	29	54	30
S-74	++	5.2	5.3	4	1	6.5	22	10
Controls								
SDS 10%		5.2	7.7	9	9	59	59	56
H_2_O		3.6	3.7	0	0	6.3	21	0
MB						10.7	29	0

MB = Marine broth.

**Table 3:** Determination of LC_50_ values for bacterial supernatants and positive controls (TBTO and CuSO_4_) on brine shrimp *Artemia franciscana* nauplii.

Supernatant	LC_50_ (µL mL^−1^)
My-30	330.8
S-69	261.8
S-74	360.4
Positive controls	LC_50_ (µg mL^−1^)
TBTO	8.7
CuSO_4_	6.2

**Table 4:** Bacterial supernatants effect on growth inhibition of marine bacteria involved in biofilm formation. Results are expressed as Minimum Inhibitory Concentration (MIC) values.

Antibacterial Activity (MIC % *v*/*v*)							
Bacterial Supernatant	Bp	Bs	Msp1	Psp1	Psp2	Ss	Vsp1
My-30	4	1	2	>10	>10	1	>10
S-69	2	1	1	>10	>10	1	>10
S-74	4	2	2	>10	>10	8	>10

Strain’s code: Bp (*Bacillus pumilus*), Bs (*Bacillus subtilis*), Msp1 (*Micrococcus* sp.1), Psp1 (*Pseudoalteromonas* sp.1), Psp2 (*Pseudoalteromonas* sp.2), Ss (*Sagittula stellata*) and Vsp1 (*Vibrio* sp.1).

## Supplementary Materials

The following are available online at https://www.mdpi.com/1422-0067/21/17/6068/s1.

## Abbreviations

AF	Antifouling
AFP	Antifouling paint
Bp	*Bacillus pumilus*
Bs	*Bacillus subtilis*
CFCS	Cell free culture supernatant
CP	Control plate
CuSO_4_	Copper sulphate
E.I.	Emulsification Index
E.S.	Emulsification stability
E24	Emulsification efficient after 24 h
Et-OH	Ethanol
LC_50_	Lethal concentration at 50%
MB	Marine broth
MIC	Minimum inhibitory concentration
Msp.1	*Micrococcus* sp. 1
My-30	*Bacillus niabensis*
OD	Optical density
Psp1	*Pseudoalteromonas* sp.1
Psp2	*Pseudoalteromonas* sp. 2
SDS	Sodium Dodecyl Sulfate
S-69	*Bacillus niabensis*
S-74	*Ralstonia* sp.
Ss	*Sagittula stellata*
TBTO	Tributyltin Oxide
UP	PC structure unpainted
Vsp1	*Vibrio* sp. 1

## References

[1] Wise J.P., Tayler J., Croom-Pereza T.J., Meaza I., Aboueissa A., López-Montalvo C.A., Martin-Brasa M., Speera R.M., Bonilla-Garzón A., Urbán J. (2019). A whale of a tale: A one environmental health approach to study metal pollution in the Sea of Cortez. Toxicol. Appl. Pharm..

[2] Becerril-Espinosa A., Freel K.C., Jensen P.R., Soria-Mercado I.E. (2013). Marine Actinobacteria from the Gulf of California: Diversity, abundance and secondary metabolite biosynthetic potential. Antonie Van Leeuwenhoek.

[3] Águila-Ramírez R.N., Hernández-Guerrero C.J., González-Acosta B., Id-Daoud G., Hewitt S., Pope J., Hellio C. (2014). Antifouling activity of symbiotic bacteria from sponge *Aplysina gerardogreeni*. Int. Biodeter. Biodegr..

[4] Sánchez-Rodríguez D., Ortiz-Aguirre I., Aguila-Ramírez R.N., Rico-Virgen E.G., González-Acosta B., Hellio C. (2018). Marine bacteria from the Gulf of California with antimicrofouling activity against colonizing bacteria and microalgae. Rev. Biol. Trop..

[5] Sriyutha M., Venugopalan V.P., Nair K.V.K., Subramonian T., Flemming H.C., Sriyutha M., Venkatesan R., Cooksey K. (2009). Larval settlement and surface: Implications in development of antifouling strategies. Marine and Industrial Biofouling.

[6] Cooney J.J., Tang R.-J. (1999). Quantifying effects of antifouling paints on microbial biofilm formation. Method. Enzymol..

[7] Eguía E., Trueba A. (2007). Application of marine biotechnology in the production of natural biocides for testing on environmentally innocuous antifouling coatings. J. Coat. Technol. Res..

[8] Yebra D.M., Kil S., Dam-Johansen K. (2004). Antifouling technology—Past, present and future steps towards efficient and environmentally friendly antifouling coatings. Prog. Org. Coat..

[9] Turner A., Singh N., Richards J.P. (2009). Bioaccessibility of metal in soils and dusts contaminated by marine antifouling paint particles. Environ. Pollut..

[10] Turner A. (2010). Marine pollution from antifouling paint particles. Mar. Pollut. Bull..

[11] Soroldoni S., Abreu F., Braga-Castro I., Duarte F.A., Lopes-Leaes P. (2017). Are antifouling paint particles a continuous source of toxic chemicals to the marine environment?. J. Hazard. Mater..

[12] Chambers L.D., Stokes K.R., Walsh F.C., Wood R.J.K. (2006). Modern approaches to marine antifouling coatings. Surf. Coat. Technol..

[13] Prakash S., Ramasubburayan R., Iyapparaj P., Arthi A.P.R., Ahila N.K., Ramkumar V.S., Immanuel G., Palavesam A. (2015). Environmentally benign antifouling potentials of Triterpenes-Glycosides from *Streptomyces fradiae*: A mangrove isolate. RSC Adv..

[14] Rivardo F., Turner R.J., Allegrone G., Ceri H., Martinotti M.G. (2009). Anti-adhesion activity of two biosurfactants produced by *Bacillus* spp. Prevents biofilm formation of human bacterial pathogens. Appl. Microbiol. Biotechnol..

[15] Sambanthamoorthy K., Feng X., Patel R., Patel S., Paranavitana C. (2014). Antimicrobial and antibiofilm potential of biosurfactants isolated from lactobacilli against multi-drug-resistant pathogens. BMC Microbiol..

[16] Polyiam P., Photisap C., Boottanun P., Wongratanacheewin S., Wongratanacheewin R., Yordpratum U. (2016). Antimicrobial and antiadhesive activities of the crude biosurfactant from *Bacillus* sp. against clinical isolates of *Acinetobacter baumannii* from Srinagarind hospital. Srinagarind Med. J..

[17] Clements T., Ndlovu T., Khan W. (2019). Broad-spectrum antimicrobial activity of secondary metabolites produced by *Serratia marcescens* strains. Microbiol. Res..

[18] Banat I.M., Makkar R.S., Cameotra S.S. (2000). Potential commercial applications of microbial surfactants. Appl. Microbiol. Biotechnol..

[19] Banat I.M., Franzetti A., Gandolfi I., Bestetti G., Martinotti M.G., Fracchia L., Smyth T., Marchant R. (2010). Microbial biosurfactants production, applications and future potential. Appl. Microbiol. Biotechnol..

[20] Lima T.M., Procopio L.C., Brandao F.D., Carvalho A.M., Totola M.R., Borges A.C. (2010). Biodegradability of bacterial surfactants. Biodegradation.

[21] Das P., Mukherjee S., Sen R. (2009). Antiadhesive action of a marine microbial surfactant. Colloid Surf. B.

[22] Dusane D.H., Matkar P., Venugopalan V.P., Kumar A.R., Zinjarde S.S. (2011). Cross-species induction of antimicrobial compounds, biosurfactants and quorum-sensing inhibitors in tropical marine epibiotic bacteria by pathogens and biofouling microorganisms. Curr. Microbiol..

[23] Dusane D.H., Pawar V.S., Nancharaiah Y.V., Venugopalan V.P., Kumar A.R., Zinjarde S.S. (2011). Anti-biofilm potential of a glycolipid surfactant produced by a tropical marine strain of *Serratia marcescens*. Biofouling.

[24] Tripathi L., Irorere V.U., Marchant R., Banat I.M. (2018). Marine derived biosurfactants: A vast potential future resource. Biotechnol. Lett..

[25] Kubicki S., Bollinger A., Katzke N., Jaeger K.-E., Loeschcke A., Thies S. (2019). Marine biosurfactants: Biosynthesis, structural diversity and biotechnological applications. Mar. Drugs.

[26] Balan S.S., Kumar C.G., Jayalakshmi S. (2016). Pontifactin, a new lipopeptide biosurfactant produced by a marine *Pontibacter korlensis* strain SBK-47: Purification, characterization and its biological evaluation. Process. Biochem..

[27] Lawrance A., Balakrishnan M., Joseph T.C., Sukumaran D.P., Valsalan V.N., Gopal D., Ramalingam K. (2014). Functional and molecular characterization of a lipopeptide surfactant from the marine sponge-associated eubacteria *Bacillus licheniformis* NIOT-AMKVO6 of Andaman and Nicobar Islands, India. Mar. Pollut. Bull..

[28] Selvin J., Sathiyanarayanan G., Lipton A.N., Al-Dhabi N.A., Valan Arasu M., Kiran G.S. (2016). Ketide synthase (KS) domain prediction and analysis of iterative type II PKS gene in marine sponge-associated actinobacteria producing biosurfactants and antimicrobial agents. Front. Microbiol..

[29] Padmavathi A.R., Pandian S.K. (2014). Antibiofilm activity of biosurfactant producing coral associated bacteria isolated from Gulf of Mannar. Indian J. Microbiol..

[30] Faÿ F., Carteau D., Linossier I., Delbury M., Vallée-Réhel K. (2013). Joint-action of antifouling substances in copper-free paints. Colloid Surf. B.

[31] Hori K., Matsumoto S. (2010). Bacterial adhesion: From mechanism to control. Biochem. Eng. J..

[32] Paul J.H., Jeffrey W.H. (1984). The effect of surfactants on the attachment of estuarine and marine bacteria to surfaces. Can. J. Microbiol..

[33] Dusane D., Zinjarde S.S., Venugopalan V.G., McLean R.J., Weber M.M., Rahman P.K. (2010). Quorum sensing: Implications on Rhamnolipid biosurfactant production. Biotech. Genet. Eng. Rev..

[34] Kosaric N. (2001). Biosurfactants and their application for soil bioremediation. Food Technol. Biotech..

[35] Singh P., Cameotra S.S. (2004). Potential applications of microbial surfactants in biomedical sciences. Trends Biotechnol..

[36] Torres-Beltrán M., Cardoso-Martínez F., Millán-Aguiñaga N., Becerril-Espinosa A., Soria-Mercado I.E. (2012). Evaluation of the Gulf of California as a potential source of bioactive marine actinobacteria. Cienc. Mar..

[37] Soon-Wo K., Seon-Young L., Byung-Yong K., Hang-Yeon W., Jung-Bong K., Seung-Joo G., Gil-Bok L. (2007). *Bacillus niabensis* sp. nov., isolated from cotton-waste composts for mushroom cultivation. Int. J. Syst. Evol. Microbiol..

[38] Miem T.M., Wiese J., Wenzel-Storjohann A., Imhoff J.F. (2015). Diversity and antimicrobial potential of bacterial isolates associated with the soft coral *Alcyonium digitatum* from the Baltic Sea. Antonie Van Leeuwenhoek.

[39] Ettoumi B., Raddadi N., Borin S., Daffonchio D., Boudabous A., Cherif A. (2009). Diversity and phylogeny of culturable spore-forming Bacilli isolated from marine sediments. J. Basic Microbiol..

[40] Kennedy J., Baker P., Piper C., Cotter P.D., Walsh M., Mooji M.J., Bourke M.B., Rea M.C., O’Connor P.M., Ross R.P. (2009). Isolation and analysis of bacteria with antimicrobial activities from the marine sponge *Haliclona simulans* collected from Irish waters. Mar. Biotechnol..

[41] Menezes C.B.A., Bonugli-Santos R.C., Miqueletto P.B., Passarini H.D.S., Justo M.R., Leal R.R., Fantinatti-Garboggini F., Oliveira V.M., Berlinck R.G.S., Sette L.D. (2010). Microbial diversity associated with algae, ascidians and sponges from the north coast of São Paulo state, Brazil. Microbiol. Res..

[42] Sayem S.A., Manzo E., Ciavatta L., Tramice A., Cordone A., Zanfardino A., de Felice M., Varcamonti M. (2011). Anti-biofilm activity of an exopolysaccharide from a sponge-associated strain of *Bacillus licheniformis*. Microb. Cell. Fact..

[43] Salanoubat M., Genin S., Artiguenave F., Gouzy J., Mangenot S., Arlat M., Billault A., Brottier P., Camus J.C., Cattolico L. (2002). Genome sequence of the plant pathogen *Ralstonia solanacearum*. Nature.

[44] Gómez M.L., Hurtado C., Dussán J., Parra J.P., Narvaéz S. (2006). Determinación de la capacidad de degradación de compuestos orgánicos persistentes por bacterias marinas aisladas de sedimentos en el Caribe colombiano. Actual. Biol..

[45] Wu P., Wang Y.S., Sun F.L., Wu M.L., Peng Y.I. (2014). Bacterial polycyclic aromatic hydrocarbon ring-hydroxylatingdioxygenases in the sediments from the Pearl River estuary, China. Appl. Microbiol. Biotechnol..

[46] Desai J.D., Banat I.M. (1997). Microbial production of surfactants and their commercial potential. Microbiol. Mol. Biol. Rev..

[47] Mondol M.A., Shin H.J., Islam M.T. (2013). Diversity of secondary metabolites from marine *Bacillus* species: Chemistry and biological activity. Mar. Drugs.

[48] Arguelles-Arias A., Ongena M., Halimi B., Lara Y., Brans A., Joris B., Fickers P. (2009). *Bacillus amyloliquefaciens* GA1 as a source of potent antibiotics and other secondary metabolites for biocontrol of plant pathogens. Microb. Cell. Fact..

[49] Youssef N.H., Dunacn K.E., Nagle D.P., Savage K.N., Knapp R.M., Mclnerney M.J. (2004). Comparison methods to detect biosurfactant production by diverse microorganism. J. Microbiol. Methods.

[50] Lara-Severino R.D., Gómez-Olivan L.M., Sandoval-Trujillo A.H., Isaac-Olive K., Ramírez-Durán N. (2017). Búsqueda de capacidad productora de biosurfactantes en actinobacterias haloalcalofilas y halotolerantes. Rev. Int. Contam. Ambient..

[51] El-Sersy N.A. (2012). Plackett-Burman design to optimize biosurfactant production by marine *Bacillus subtilis* N10. Rom. Biotech. Lett..

[52] Fonseca de Faria A., Teodoro-Martinez D.S., de Oliveira G.N., Vaz B.G., Serrano-Silva I., Garcia J.S., Tótola M.R., Eberlin M.N., Grossman M., Alvez O.L. (2011). Production and structural characterization of surfactin (C14/ Leu7) produced by *Bacillus subtilis* isolate LSFM-05 grown on raw glycerol from the biodiesel industry. Process Biochem..

[53] Youcef-Ali M., Kacem N., Dehimat L., Ait A., Destain J., Thornart P. (2013). Selection of an Antifungal *Bacillus niabensis* from Algerian salt soil and study of its potential of surfactin production. Sch. J. Agric. Sci..

[54] Plaza G.A., Lukasik K., Wypych J., Nalecz-Jawecki G., Berry C., Brigmon R.L. (2008). Biodegradation of crude oil and distillation products by biosurfactant-producing bacteria. Pol. J. Environ. Stud..

[55] Plaza G.A., Zjawiony I., Banat I.M. (2006). Use of different methods for detection of thermophilic biosurfactant producing bacteria from hydrocarbon-contaminated and bioremediated soils. J. Pet. Sci. Eng..

[56] Sudip K.S., Bharat B., Ashvini C., Rakesh K.J. (2000). Chemotaxis of a *Ralstonia* sp. SJ98 toward different nitroaromatic compounds and their degradation. Biochem. Biophys. Res..

[57] Trefault N., De la Iglesia R., Molina A.M., Manzano M., Ledger T., Perez-Pantoja D., Sánchez M.A., Stuardo M., Gonzalez B. (2004). Genetic organization of the catabolic plasmid pJP4 from *Ralstonia eutropha* JMP134 (pJP4) reveals mechanisms of adaptation to chloroaromatic pollutants and evolution of specialized chloroaromatic degradation pathways. Environ. Microbiol..

[58] Chen W.M., Chang J.S., Wu C.H., Chang S.C. (2004). Characterization of phenol and trichloroethene degradation by the rhizobium *Ralstonia taiwanensis*. Res. Microbiol..

[59] Wang Y.D., Dong X.J., Wang X., Hong Q., Jiang X., Li S.P. (2007). Isolation of phenol-degrading bacteria from natural soil and their phylogenetic analysis. China Environ. Sci..

[60] Adebusoye S.A., Picardal F.W., Ilori M.O., Amund O.O., Fuqua C. (2008). Characterization of multiple novel aerobic polychlorinated biphenyl (PCB)-utilizing bacterial strains indigenous to contaminated tropical African soils. Biodegradation.

[61] Waldau D., Mikolasch A., Lalk M., Schauer F. (2009). Derivatization of bioactive carbazoles by the biphenyl-degrading bacterium *Ralstonia* sp. strain SBUG 290. Appl. Microbiol. Biotechnol..

[62] Bucheli-Witschel M., Hafner T., Ruegg I., Egli T. (2009). Benzene degradation by *Ralstonia pickettii* PKO1 in the presence of the alternative substrate succinate. Biodegradation.

[63] Li J., Liu J., Shen W., Zhao X., Hou Y., Cao H., Cui Z. (2010). Isolation and characterization of 3,5,6-trichloro-2-pyridinol-degrading *Ralstonia* sp. strain T6. Bioresour. Technol..

[64] Fernández-Calienes V.A., Mendiola-Martínez J., Monzote-Fidalgo L., García-Parra M., Sariego-Ramos I., Acuña-Rodríguez D., Scull L.R., Gutiérrez-Gaitén Y. (2009). Evaluación de la toxicidad de extractos de plantas cubanas con posible acción antiparasitaria utilizando larvas de *Artemia salina*. Rev. Cuba. Med. Trop..

[65] Amara I., Miled W., Slama R.B., Ladhari N. (2018). Antifouling processes and toxicity effect of antifouling paints on marine environment. A review. Environ. Toxicol. Pharmacol..

[66] Othmani A., Bunet R., Bonnefont J.L., Briand J.F., Culioli G. (2016). Settlement inhibition of marine biofilm bacteria and barnacle larvae by compounds isolated from the Mediterranean brown alga, *Taonia atomaria*. J. App. Phycol..

[67] Fernandes P.A.V., de Arruda I.R., Botelho A.F.A., de Araújo A.A., Souto A.M., Azevedo E. (2007). Antimicrobial activity of surfactants produced by *Bacillus subtilis* against multidrug-resistance bacteria. Braz. J. Microbiol..

[68] Wong J.H., Hao J., Cao Z., Qiao M., Xu H., Bai Y., Ng T.B. (2008). An antifungal protein from *Bacillus amyloliquefaciens*. J. Appl. Microbiol..

[69] Hong-Mei X., Yan-Jun R., Ming-Xin Z., Bo S., Zhen-Ming C. (2014). Antibacterial activity of the lipopetides produced by *Bacillus amyloliquefaciens* M1 against multidrug-resistant *Vibrio* spp. isolated from diseased marine animals. Appl. Microbiol. Biot..

[70] Vatsa P., Sanchez L., Clement C., Baillieul F., Dorey S. (2010). Rhamnolipid biosurfactants as new players in animal and plant defense against microbes. Int. J. Mol. Sci..

[71] Mah T.F.C., O’Toole G. (2001). Mechanisms of biofilm resistance to antimicrobial agents. Trends Microbiol..

[72] (2015). International Antimicrobial Council. https://amcouncil.org/antimicrobial-coatings-market-expected-to-reach-4520-3-million-by-2020/.

[73] Soares da Silva R.C.F., de-Almeida D.G., Brasileiro P.P.F., Rufino R.D., de Luna J.M., Sarubbo L.A. (2019). Production, formulation and cost estimation of a commercial biosurfactant. Biodegradation.

[74] Santos D.K., Rufino R.D., Luna J.M., Santos V.A., Sarubbo L.A. (2016). Biosurfactants: Multifunctional biomolecules of the 21st Century. Int. J. Mol. Sci..

[75] Prado A.A.O.S., Santos B.L.P., Vieira I.M.M., Ramos L.C., de Souza R.R., Silva D.P., Ruzenea D.S. (2019). Evaluation of a new strategy in the elaboration of culture media to produce surfactin from hemicellulosic corncob liquor. Biotechnol. Rep..

[76] Kim M., Park J.M., Um H.J., Lee K.H., Kim H., Min J., Kim Y.H. (2011). The antifouling potentiality of galactosamine characterized from *Vibrio vulnificus* exopolysaccharide. Biofouling.

[77] Satheesh S., Soniamby A.R., Sunjaiy-Shankar C.V., Punitha S.M.J. (2012). Antifouling activities of marine bacteria associated with sponge (*Sigmadocia* sp.). J. Ocean Univ. China.

[78] Jin C., Xin X., Yu S., Qiu J., Miao L., Feng K., Zhou X. (2014). Antidiatom activity of marine bacteria associated with sponges from San Juan Island, Washington. World J. Microb. Biot..

[79] Giri S., Ryu E.C., Sukumaran V., Parka S.C. (2019). Antioxidant, antibacterial, and anti-adhesive activities of biosurfactants isolated from *Bacillus* strains. Microb. Pathog..

[80] Singh N., Turner A. (2009). Trace metals in antifouling paint particles and their heterogeneous contamination of coastal sediments. Mar. Pollut. Bull..

[81] Acevedo M.S., Puentes C., Carreño K., León J.G., Stupak M., García M., Pérez M., Blustein G. (2013). Antifouling paints based on marine natural products form Colombian Caribbean. Int. Biodeter. Biodegr..

[82] Soliman Y.A., Ibrahim M.A., Tadros H.R.Z., Abou-Taleb A.E.A., Moustafa A.H., Hamed M.A. (2016). Antifouling and antibacterial activities of marine bioactive compounds extracted from some Red sea cucumber. Int. J. Contemp. App. Sci..

[83] Burgess J.B., Boyd K.G., Armstrong E., Jiang Z., Yan L., Berggren M., May U., Pisacane T., Granmo A., Adams D.R. (2003). The development of a marine natural products-based antifouling paint. Biofouling.

[84] Palanichamy S., Subramanian G. (2016). Antifouling properties of marine bacteriocin incorporated epoxy based paint. Prog. Org. Coat..

[85] Armstrong E., Boyd K.G., Pisacane A., Peppiatt C.J., Burgess J.G. (2000). Marine microbial natural products in antifouling coatings. Biofouling.

[86] Dobretsov S., Abed R.M.M., Voolstra C.R. (2013). The effect of surface colour on the formation of marine micro and macrofouling communities. Biofouling.

[87] Li Y.F., Guo X.P., Chen Y.R., Ding D.W., Yang J.L. (2017). Comparative analysis of biofilm community on different coloured substrata in relation to mussel settlement. J. Mar. Biol. Assoc. UK.

[88] Schumacher J.F., Carman M.L., Estes T.G., Feinberg A.W., Wilson L.H., Callow M.E., Callow J.A., Finlay J.A., Brennan B. (2007). Engineered antifouling microtopographies—Effect of feature size, geometry, and roughness on settlement of zoospores of the green alga *Ulva*. Biofouling.

[89] Vesco S., Aversa C., Puopolo M., Barletta M. (2019). Advances in design and manufacturing of environmentally friendly and biocide-free antifouling/foul-release coatings: Replacement of fluorinate species. J. Coat. Technol. Res..

[90] Análisis de Costo Beneficio, Protocolo de Detección Temprana y Respuesta Rápida y Evaluación de Impactos Económicos Para Tunicados en Baja California. https://www.biodiversidad.gob.mx/media/1/especies/Invasoras/files/comp1/Analisis_costo_beneficio_control_de_tunicados.zip.

[91] Sánchez-Lozano I., Hernández-Guerrero C.J., Muñoz-Ochoa M., Hellio C. (2019). Biomimetic approaches for the development of new antifouling solutions: Study of incorporation of macroalgae and sponge extracts for the development of new environmentally-friendly coatings. Int. J. Mol. Sci..

[92] Duncan K., Haltli B., Gill K.A., Kerr R.G. (2014). Bioprospecting from marine sediments of New Brunswick, Canada: Exploring the relationship between total bacterial diversity and actinobacteria diversity. Mar. Drugs.

[93] Stach J.E., Bathe S., Clapp J.P., Burns R.G. (2001). PCR-SSCP comparison of 16S rDNA sequence diversity in soil DNA obtained using different isolation and purification methods. FEMS Microbiol. Ecol..

[94] Mulligan C., Cooper D., Neufeld R. (1984). Selection of microbes producing biosurfactants in media without hydrocarbons. J. Ferment. Technol..

[95] Morikawa M., Hirata Y., Imanaka T. (2000). A study on the structure-function relationship of lipopeptide biosurfactants. Biochim. Biophys. Acta.

[96] Das M., Das S.K., Mukherjee R.K. (1998). Surface active properties of the culture filtrates of a *Micrococcus* species grown on n-alkanes and sugars. Bioresour. Technol..

[97] Meyer B.N., Ferrigni N.R., Putnam J.E., Jacobsen L.B., Nichols D.E., McLaughlin J.L. (1982). Brine Shrimp: A convenient general bioassay for active plant constituents. Planta Med..

[98] Hellio C., Trepos R., Aguila-Ramírez R.N., Hernández-Guerrero C.J., Stengel D.B., Connan S. (2015). Protocol for assessing antifouling activities of macroalgal extracts. Natural Products from Marine Algae: Methods and Protocols (Methods in Molecular Biology).

