# Supplementary figures and images for: Exploring Antifouling Activity of Biosurfactants Producing Marine Bacteria Isolated from Gulf of California
Source: Int J Mol Sci. 2020 Aug 23;21(17):6068. doi: 10.3390/ijms21176068 (PMC7504147; doi:10.3390/ijms21176068)

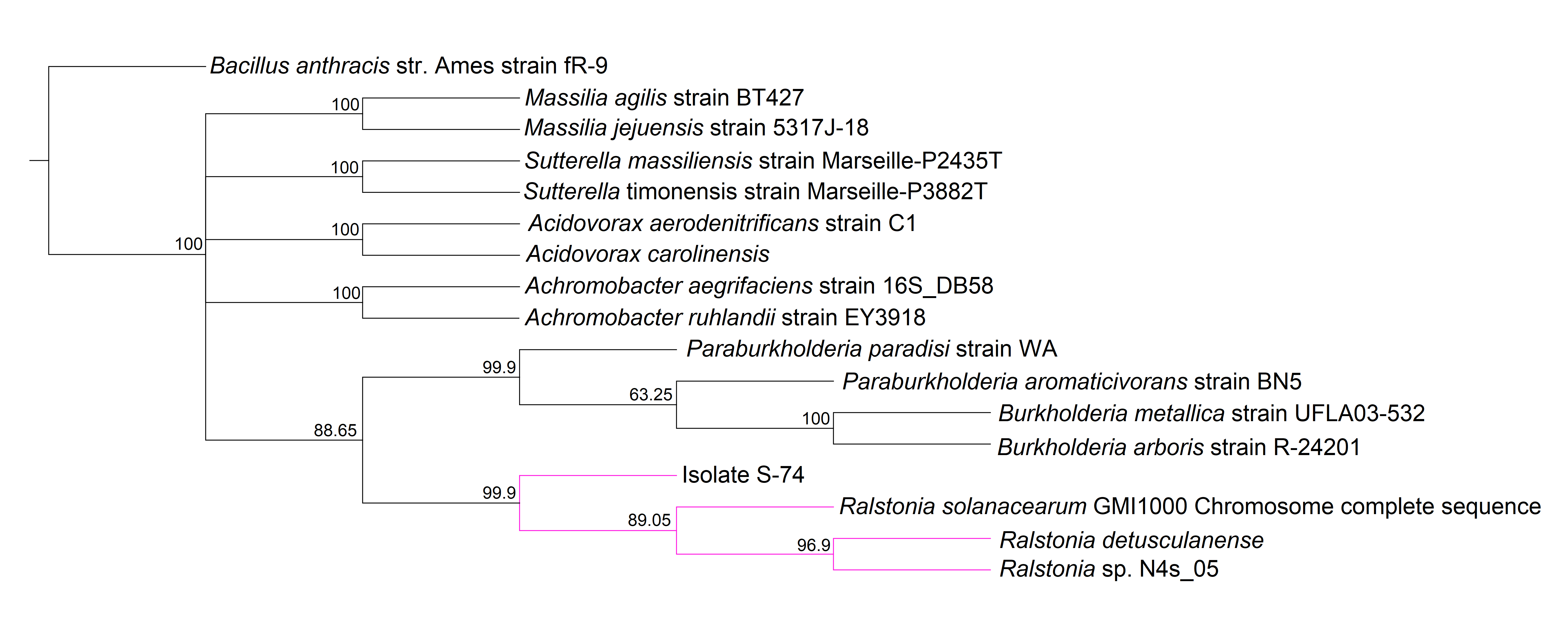

Supplement: Supplementary file 1 [file ijms-21-06068-s001.zip › S3.png]

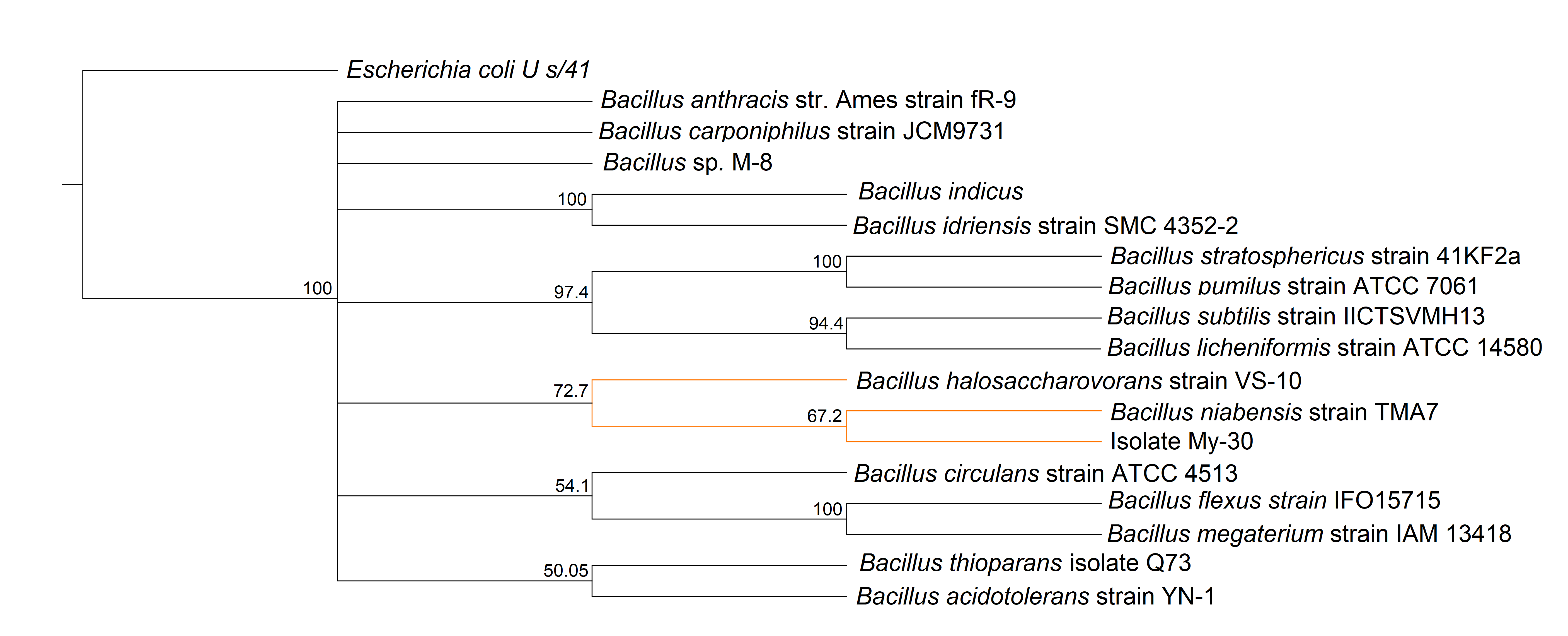

Supplement: Supplementary file 1 [file ijms-21-06068-s001.zip › S1.png]

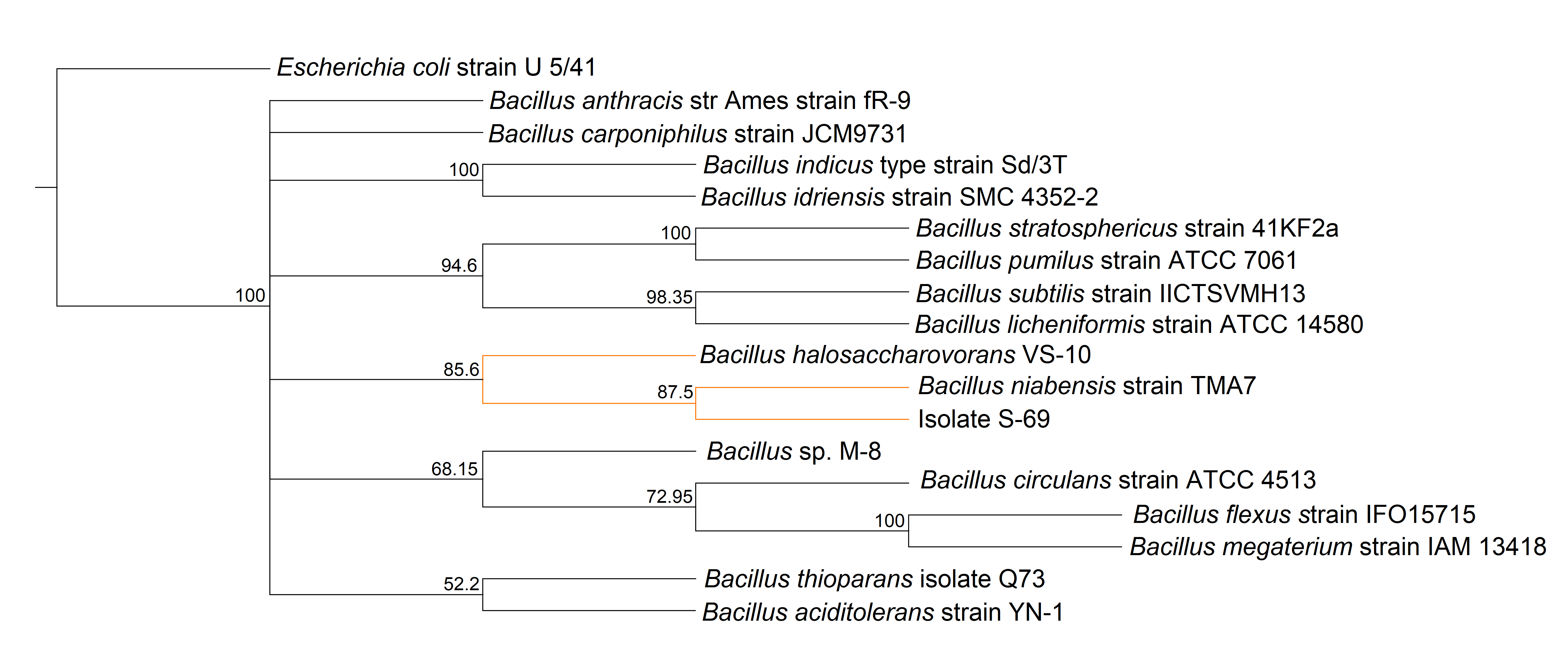

Supplement: Supplementary file 1 [file ijms-21-06068-s001.zip › S2.png]
